# SMARCA4 regulates SMARCAD1 expression for toleration of replication stress in non-small cell lung cancer

**DOI:** 10.20407/fmj.2025-017

**Published:** 2025-11-05

**Authors:** Patinya Sawangsri, Siripan Limsirichaikul, Toshiyuki Takeuchi, Yasuyoshi Mizutani, Dat Quoc Tran, Taisuke Kajino, Motoshi Suzuki, Atsuko Niimi

**Affiliations:** 1 Department of Molecular Oncology, Fujita Health University, School of Medicine, Toyoake, Aichi, Japan; 2 Division of Molecular Diagnostics, Aichi Cancer Center Research Institute, Nagoya, Aichi, Japan

**Keywords:** NSCLC, Replication stress, SMARCA4, SMARCAD1

## Abstract

**Objectives:**

SMARCA4, a core component of the SWI/SNF chromatin remodeling complex, is frequently mutated in non-small cell lung cancer (NSCLC). SMARCA4-deficient cancer cells are associated with increased replication stress, one of the major causes of genomic instability, which may lead to cancer. SMARCAD1, a chromatin remodeler, is known as replication fork progressor, and SMARCAD1 dysregulation is also closely related to cancer development. This study aimed to investigate the role of the SMARCA4-SMARCAD1 axis in the toleration of replication stress in NSCLC, focusing on the regulatory relationship between SMARCA4 and SMARCAD1 during replication stress conditions.

**Methods:**

Human NSCLC cell lines (Calu-6, NCI-H1975, Calu-1, and NCI-H460) were used for experiments. SMARCA4 and SMARCAD1 expression levels were analyzed by quantitative RT-PCR and immunoblotting. Transcriptional regulation of SMARCAD1 was analyzed by chromatin immunoprecipitation assay. Immunofluorescent analysis was performed to assess SMARCAD1 accumulation at stalled replication forks. Clonogenic assays were conducted to evaluate the roles of SMARCA4 and SMARCAD1 in cell survival.

**Results:**

SMARCAD1 was highly expressed in SMARCA4-depleted cells under replication stress. Immunofluorescent analysis revealed significant accumulation of SMARCAD1 at stalled replication forks in SMARCA4-depleted cells. Chromatin immunoprecipitation assays demonstrated that SMARCA4 bound to the transcriptional regulatory region of *SMARCAD1*, and that this efficacy was decreased under replication stress, suggesting that SMARCA4 is a transcriptional suppressor of *SMARCAD1*. In a clonogenic analysis either SMARCA4 or SMARCAD1 is required for cell survival.

**Conclusions:**

The SMARCA4-SMARCAD1 axis is a novel mechanism that provides tolerance for replication stress.

## Introduction

The *SMARCA4* (SWI/SNF-related, matrix associated, actin-dependent regulator of chromatin, subfamily A, member 4) gene encodes SMARCA4/BRG1, an ATP-dependent catalytic subunit of the SWI/SNF chromatin remodeling complex, which modulates chromatin architecture and regulates gene transcription, as well as DNA replication and DNA repair.^1,2^ It has been shown that *SMARCA4* is frequently mutated in non-small cell lung cancer (NSCLC) (~10%), with the majority of those mutations loss-of-function type.^3,4^ *SMARCA4*-deficient patients show resistance to chemotherapy and generally have a poor prognosis.^5,6^ These findings indicate that *SMARCA4* is a tumor suppressor gene. However, contrasting results have shown that the expression level of SMARCA4 is frequently upregulated in various types of cancer and a high SMARCA4 expression level is also associated with poor patient prognosis,^7^ suggesting a tumor promotion role for SMARCA4.

SMARCAD1 (SWI/SNF-related, matrix associated, actin-dependent regulator of chromatin, containing DEAD/H box1), a member of the DEAD/H box-containing group of helicases, is known to regulate gene transcription as a chromatin remodeler,^8^ while it also has roles in DNA double-strand break repair^9,10^ and mismatch repair.^11,12^ Additionally, high levels of expression are often noted in breast cancer,^13,14^ hepatocellular carcinoma,^15^ and pancreatic cancer,^16^ suggesting that SMARCAD1 dysregulation is closely related to cancer development.

DNA replication is a well-organized and accurate process. Nevertheless, under some circumstances, such as oncogene-driven forced replication or replication of a secondary DNA structure like the G4-quadruplex, the process becomes perturbed, and replication stress is accumulated.^17–20^ In the presence of replication stress, replication forks become stalled and stabilized by remodeling processes, then the stressed forks are restarted or bypassed via DNA damage tolerance and repair pathways, such as translesion synthesis, homologous recombination, and break-induced replication as well as others.^21–24^ Insufficient resolution of replication stress is considered one of the major sources of genomic instability and results in carcinogenesis.^25–27^

From the viewpoint of DNA replication, loss of SMARCA4 may alter proper chromatin organization^28^ and increase the heterochromatin region, one of the causes of replication stress.^18,29,30^ SMARCA4 suppresses R-loop-mediated transcription-replication conflict, which induces replication stress,^31^ resulting in efficient fork progression.^32^ SMARCAD1 interacts with proliferating cell nuclear antigen (PCNA), a clamp in the DNA replication machinery, for the progression of forks.^33^ SMARCAD1 is also involved in *de novo* nucleosome assembly and replication-coupled heterochromatin organization.^34–36^ Therefore, both SMARCA4 and SMARCAD1 likely function to suppress replication stress, and co-operatively act for replication progress, though the underlying mechanisms have yet to be elucidated.

Reported here are findings showing upregulation of SMARCAD1 in SMARCA4-depleted cells in the presence of hydroxyurea (HU), a replication stress inducer. SMARCA4 was shown to bind to the transcription regulatory region of *SMARCAD1*, while that binding was reduced with HU treatment. Furthermore, SMARCAD1 recruitment at stalled replication forks was significantly increased in SMARCA4-depleted cells. It is thus proposed that the SMARCA4-SMARCAD1 axis is a novel mechanism that provides tolerance for replication stress in NSCLC cells.

## Methods

### Cell cultures, antibodies, and chemical reagents

Calu-6, NCI-H1975, Calu-1, and NCI-H460 cells were provided by Professor Takashi Takahashi at Nagoya University School of Medicine. The cells were cultured and maintained in RPMI1640 with L-glutamine (FUJIFILM, Osaka, Japan), supplemented with 5% fetal bovine serum (Thermo Fisher Scientific, Massachusetts, USA). We confirmed that Calu-6 (cell number: KBN0546-03), NCI-H1975 (cell number: KBN0613-06), Calu-1 (cell number: KBN0613-09), and NCI-H460 (cell number: KBN0546-02) cells were the same as those registered in the JCRB Cell Bank. All cell lines were demonstrated to be free from mycoplasma infection by using a MycoAlert kit (Lonza, Basel, Switzerland). The antibodies and chemical reagents used in this study are presented in Table S1.

### siRNA knockdown

Cells were transfected with 20 nM of each siRNA duplex using the Neon Transfection System (Thermo Fisher Scientific) following the manufacturer’s instructions and cultured for 72 hours. The sequences of the siRNA oligonucleotides used in this study are shown in Table S2.

### Quantitative RT-PCR

Total RNA was extracted using an miRNeasy kit (Qiagen, Venlo, Netherlands), followed by cDNA synthesis with a SuperScript VILO cDNA Synthesis Kit (Thermo Fisher Scientific), following the manufacturers’ instructions. SYBR green quantitative RT-PCR analysis was performed using a QuantiTect SYBR Green PCR kit (Qiagen) and Rotor Gene Q system (Qiagen), with some modifications, as previously reported.^37^ Briefly, a 20 μL reaction mixture containing an equal volume of cDNA, 0.3 μM each of forward and reverse primers (Table S2), and 10 μL of PCR Master Mix was used. Ct values were normalized to those of 18S (ΔCt), and the average ΔΔCt values were calculated by normalization to the ΔCt value of siCTRL-treated cells, as previously described.^37^ Experiments were performed three or more times (indicated in the respective figure legends), with values shown as the mean with standard deviation (SD).

### Immunoblotting

Cells were washed in PBS, and Laemmli buffer (125 mM Tris-HCl, pH 6.8, 4% SDS, 10% 2-mercaptoethanol, 20% glycerol, 0.004% bromophenol blue) was added to the plates to obtain whole cell extracts. Triton X-100 extraction was performed as previously described.^38^ Cells were harvested by scraping and were then boiled and sonicated. Protein samples were separated by SDS-PAGE gel electrophoresis and then transferred to a PVDF membrane (Immobilon-P, Merck Millipore, Massachusetts, USA). The membrane was blocked in TBS containing 0.05% Tween 20 (TBS-T) and 5% skim milk. Immunoblotting was performed with anti-SMARCA4, anti-histone H3, anti-PCNA, or anti-SMARCAD1 antibodies. Signals were detected with use of a LuminoGraph I camera (ATTO, Tokyo, Japan). The experiments were performed at least twice.

### Chromatin immunoprecipitation (ChIP) assay

ChIP assays were performed using a SimpleChIP Plus Sonication Chromatin IP kit (Cell Signaling Technology, Massachusetts, USA), following the manufacturer’s instructions. Calu-6 cells were cultured for 24 hours with or without 1 mM HU and then used for cross-linked immunoprecipitation of chromatin with anti-SMARCA4 (Abcam, Cambridge, UK) and a Dynabeads Co-immunoprecipitation kit (Thermo Fisher Scientific). qPCR was performed as described above. Primers used to amplify the *SMARCAD1* regulatory region are listed in Table S2. These experiments were performed in triplicate. Values are shown as the mean with SD.

### Immunofluorescent analysis

siRNA-transfected cells were seeded onto coverslips (MATSUNAMI, Osaka, Japan) and cultured for 72 hours. Cells were then treated with Triton X^39^ and fixed using PBS containing 3% paraformaldehyde and 2% sucrose for 10 minutes, followed by blocking with PBS containing 0.1% Tween 20 (PBS-T) and 1% bovine serum albumin (BSA). Next, the cells were incubated with a primary antibody in the same buffer for 2 hours, washed with PBS-T, and incubated with an appropriate secondary antibody conjugated with either Alexa Fluor 488 or 568 and DAPI in 1% BSA/PBS-T for 1 hour. Cells were washed with PBS-T and PBS and then mounted with Fluoromount mounting medium (Diagnostic Biosystems, California, USA). For image acquisition, an LSM-710 confocal microscope (Zeiss, Oberkochen, Germany) equipped with an x63 objective lens and the ZEN 2.6 image acquisition software package (Zeiss) were used.

### Co-localization of RPA and SMARCAD1

Surface rendering of SMARCAD1 and determination of the center of fluorescence intensity of RPA were performed using the Imaris software package, version 9.8.2 (Oxford Instruments, Abingdon, UK). Co-localization scores were determined using more than 60 cells. The experiments were performed in duplicate. Values are shown as the mean with SD.

### Colony formation assay

Seventy-two hours after siRNA transfection, Calu-6 and NCI-H1975 cells were cultured in medium containing HU for 36 and 48 hours, respectively. Cells were washed three times with PBS and fresh medium was added. After 10–14 days of incubation, colonies were stained with Gram’s crystal violet solution (Merck) in 25% methanol for 15 minutes and counted.

### Statistical analyses

Data are expressed as the mean±SD, and n indicates the number of independent experiments. For paired samples, statistical significance was determined using Welch’s t-test and one-way ANOVA, and Tukey’s test was used for paired and multiple comparisons. *P*-values less than 0.05 indicated statistical significance.

### Availability of data and materials

The LUAD dataset (GSE11969) analyzed in the current study is publicly available in the Gene Expression Omnibus (GEO) repository.^40^

## Results

### Loss of SMARCA4 induced replication fork stalling in NSCLC cell lines

**Figure 1:**
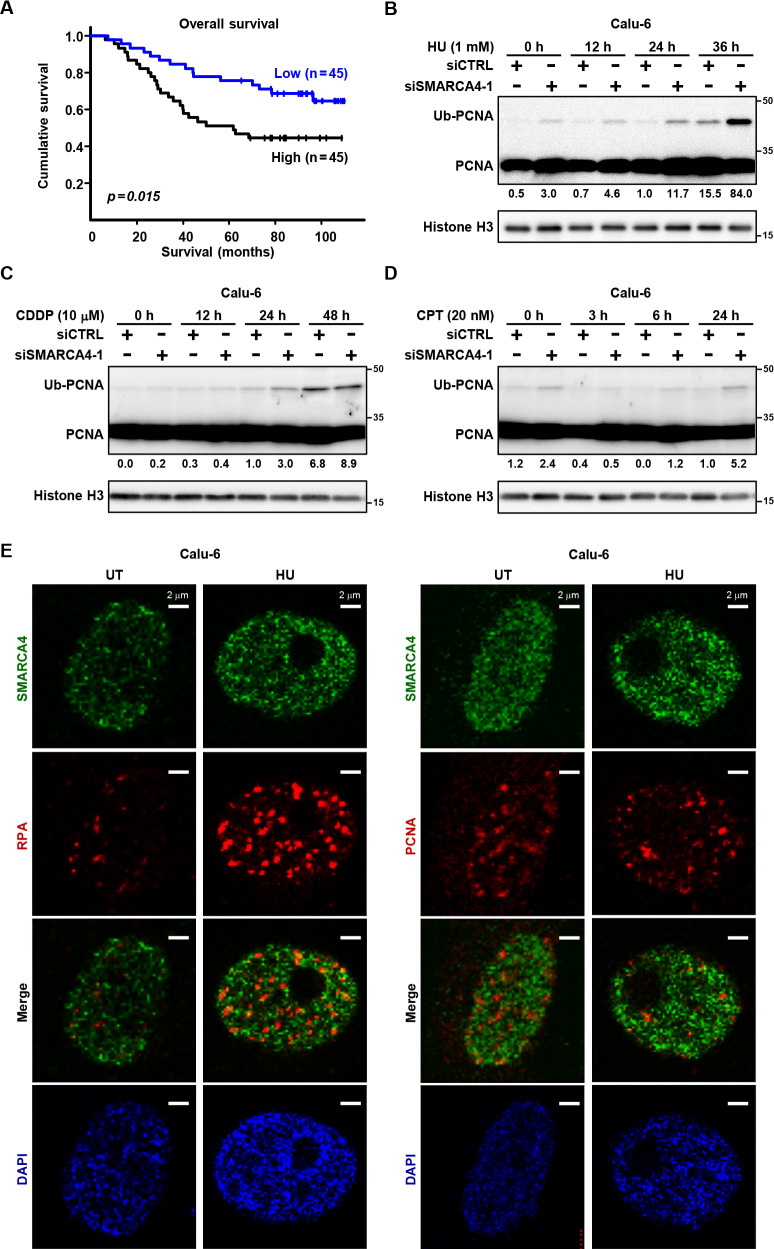
SMARCA4 is associated with prognosis and required for fork progression (A) Overall survival curves (Kaplan–Meier analysis). Lung adenocarcinoma (LUAD) patients (n=90) were classified into high and low SMARCA4 expression groups using the median value of SMARCA4 expression as the threshold. A log-rank test was used to determine *p*-values. (B–D) Calu-6 cells transfected with siCTRL or siSMARCA4-1 were analyzed for PCNA ubiquitination (Ub-PCNA) following treatment with 1 mM hydroxyurea (HU) (B), 10 μM cisplatin (CDDP) (C), or 20 nM camptothecin (CPT) (D). Relative values to histone H3 are indicated. (E) Calu-6 cells were treated with 1 mM HU (HU) or untreated (UT) for 24 hours, and immunofluorescence was performed. Representative images of nuclear localization of SMARCA4 (green), with RPA (red) or PCNA (red) are shown. Scale bar, 2 μm. Experiments shown in (B–E) were performed at least twice, with similar results obtained.

Loss-of-function mutations in *SMARCA4* genes are present in approximately 10% of NSCLC cases, and *SMARCA4*-deficient patients are reported to have a poor prognosis.^3–6^ High SMARCA4 expression was shown to result in alteration of gene expression patterns and has been associated with poor prognosis in various types of cancer. Therefore, tight regulation of SMARCA4 expression may be required to suppress cancer development and/or progression. The present analysis conducted using an LUAD dataset (GSE11969) also showed poor prognosis for patients with a high level of SMARCA4 expression (Figure 1A).

To examine the role of SMARCA4 in replication stress, we depleted SMARCA4 in Calu-6 cells by siRNA and evaluated the extent of stalled replication forks by assessing PCNA ubiquitination. In the absence of DNA replication stress, SMARCA4 knockdown, performed with use of two independent siRNAs, induced a moderate increase in PCNA ubiquitination level; a greater increase was noted in the presence of HU (Figure 1B; Figure S1A, B). These results indicate that SMARCA4 may function to release replication stress. Additionally, these findings are consistent with previous DNA fiber analysis findings showing that SMARCA4 depletion induced fork speed slowing.^41^ Relatively weak inducers of PCNA ubiquitination, such as cisplatin and camptothecin,^42,43^ showed reduced effects compared with HU (Figure 1C, D; Figure S1C, D). Similar results were observed with NCI-H1975 cells (Figure S1E–H).

SMARCA4 has been reported to localize with replication factors^32^ and directly stabilize replication forks under replication stress.^31^ To analyze SMARCA4 localization in NSCLC cells, immunofluorescent analysis was performed. As shown in Figure 1E, SMARCA4 was localized in a pan-chromatin manner and did not particularly accumulate with either PCNA or RPA, which are located at replication forks and single-stranded DNA regions in front of the forks induced by replication stress, respectively.^25^ These results suggest that the major pathway for SMARCA4 in the NSCLC cell line Calu-6 to suppress replication stress may not be through co-localization at the replication forks, but rather by another route.

### Depletion of SMARCA4 leads to upregulation of SMARCAD1

**Figure 2:**
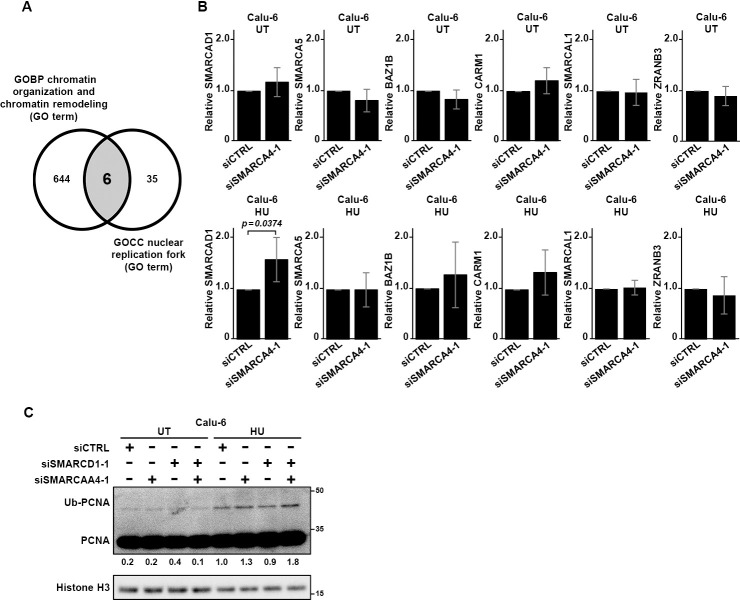
Gene screening for fork progression under replication stress in SMARCA4-depleted cells (A) Venn diagram with the Gene Ontology (GO) terms GOCC, for the nuclear replication fork in the cellular component, and GOBP, for chromatin organization/remodeling in the biological process. (B) mRNA levels of six candidate genes, *SMARCAD1*, *SMARCA5*, *BAZ1B*, *CARM1*, *SMARCAL1*, and *ZRANB3*, were determined in Calu-6 cells treated with 1 mM HU (HU) or untreated (UT) using qPCR. *P*-values were calculated from five independent experiments. (C) Calu-6 cells transfected with siCTRL, siSMARCAD1-1, and/or siSMARCA4-1 with or without treatment with 1 mM HU were analyzed for PCNA ubiquitination (Ub-PCNA). Relative values to histone H3 are indicated.

To elucidate the mechanisms involved in overcoming stalled fork progression, *in silico* analysis was performed using two filters noted by the Gene Ontology (GO) terms GOCC, for the nuclear replication fork in the cellular component (Table S3), and GOBP, for chromatin organization/remodeling in the biological process (Table S4). From these results, the *SMARCAD1*, *SMARCA5*, *BAZ1B*, *CARM1*, *SMARCAL1*, and *ZRANB3* genes were chosen (Figure 2A). qPCR analysis showed that under HU-treated conditions, *SMARCAD1* mRNA level was significantly greater in SMARCA4-depleted Calu-6 cells compared with control cells (*p*=0.0374) (Figure 2B). Similar results were obtained with NCI-H1975 cells (*p*=0.0256) (Figure S2A). The other five genes did not show elevation in both Calu-6 and NCI-H1975 cells under HU-treated conditions.

Double depletion of SMARCA4 and SMARCAD1 from Calu-6 cells induced more PCNA ubiquitination than SMARCA4 single depletion under HU treatment (Figure 2C). Similar results were obtained with NCI-H1975 cells (Figure S2B). These findings suggest that SMARCA4 and SMARCAD1 may cooperatively contribute to restoring replication fork progression.

### Regulation of SMARCAD1 expression by SMARCA4 in Calu-6 cells

**Figure 3:**
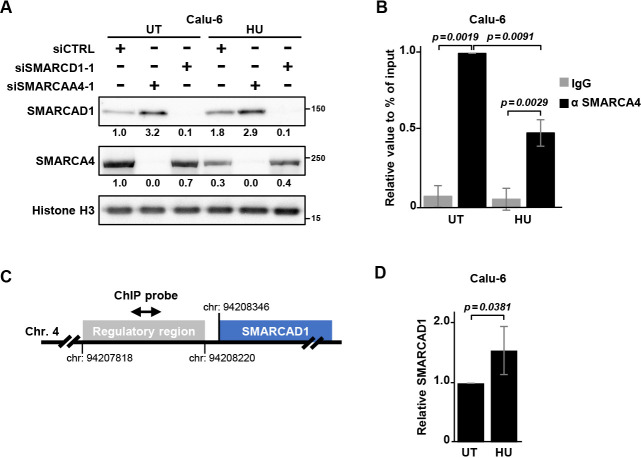
SMARCAD1 expression is regulated by SMARCA4 (A) Immunoblotting of Calu-6 cells under the indicated knockdown conditions. SMARCA4 depletion resulted in upregulation of SMARCAD1 under both treatment with 1 mM HU (HU) and untreated (UT) conditions. Relative values to histone H3 are indicated. (B) ChIP assays were performed in Calu-6 cells with anti-SMARCA4 antibody and primers for the transcriptional regulatory region of *SMARCAD1*. Values relative to the input are shown as the mean±SD of experiments performed three times. (C) Illustration of the analyzed ChIP region in *SMARCAD1*. The region located in a putative regulatory element identified by ChIP-seq analysis of SMARCA4 in MCF-7 cells (ReMap Atlas of Regulatory Regions, GSE123284 SMARCA4, MCF-7). (D) mRNA levels of *SMARCAD1* in Calu-6 cells treated with 1 mM HU (HU) or untreated (UT) cells were determined using qPCR. *P*-values were calculated from five independent experiments.

Our results above indicated SMARCA4 and SMARCAD1 may function in restoring replication fork progression, and a previous study indicated that SMARCAD1 is a replication fork progression factor.^33^ We next performed experiments to analyze the relationship between SMARCA4 and SMARCAD1. In SMARCA4-depleted cells treated with HU, SMARCAD1 protein levels were increased (Figure 3A; Figure S3A, B), while SMARCA4 levels were similar between the control and SMARCAD1-depleted cells. Notably, SMARCAD1 was also increased in SMARCA4-depleted cells in the absence of HU. These results suggest that SMARCAD1 expression is regulated not only at the transcription step but also possibly at the translation and/or protein degradation steps.

We also examined SMARCA4 and SMARCAD1 protein levels in three other NSCLC cell lines after knockdown of SMARCAD1 or SMARCA4 (Figure S3C–E). In SMARCA4-depleted NCI-H1975 cells treated with HU, SMARCAD1 expression was increased compared with levels in siCTRL cells, consistent with the observation in Calu-6 cells (Figure S3C); this result was not seen in Calu-1 or NCI-H460 cells (Figure S3D, E). These results indicate that multiple regulation mechanisms are employed downstream of SMARCA4.

The SWI/SNF complex has a role as a transcription factor, and the transcriptional regulatory region of *SMARCAD1* has been reported to contain a putative SWI/SNF binding site.^44,45^ We next performed ChIP assays. The results showed binding of SMARCA4 to the transcriptional regulatory region of *SMARCAD1* in Calu-6 cells, and the binding was decreased in cells treated with HU (Figure 3B, C). The reduced binding of SMARCA4 to the transcriptional regulatory region of *SMARCAD1* in HU might induce the elevation of *SMARCAD1* mRNA level (Figure 3D).

### Loss of SMARCA4 induced the accumulation of SMARCAD1 at stalled forks in the presence of replication stress

SMARCAD1 was previously reported to be a putative SMARCA4 binding partner,^34^ suggesting the possibility that the two proteins co-localize and cooperate in chromatin organization to suppress replication stress. We therefore performed immunofluorescent analysis to visualize the localization of SMARCA4 and SMARCAD1 in Calu-6 cells. The results did not show co-localization of the two proteins (Figure 4A). Another possibility is that SMARCA4 influences SMARCAD1 localization at replication forks. Co-staining of SMARCAD1 and RPA revealed several SMARCAD1 foci co-localized with RPA in SMARCA4-depleted cells (Figure 4B), with numbers greater than those observed in control cells (Figure S4A, B). Similar results were obtained with NCI-H1975 cells (Figure S4C, D). These findings suggest that upregulated SMARCAD1 accumulates at the single strand region of stalled forks in Calu-6 and NCI-H1975 cells. Previous studies reported localization of SMARCAD1 in replications forks in MRC5 human fibroblast cells,^33,34^ and a physical interaction between SMARCAD1 and RPA has also been reported.^46^

### The SMARCA4-SMARCAD1 axis is required for fork progression to overcome replication stress

**Figure 5:**
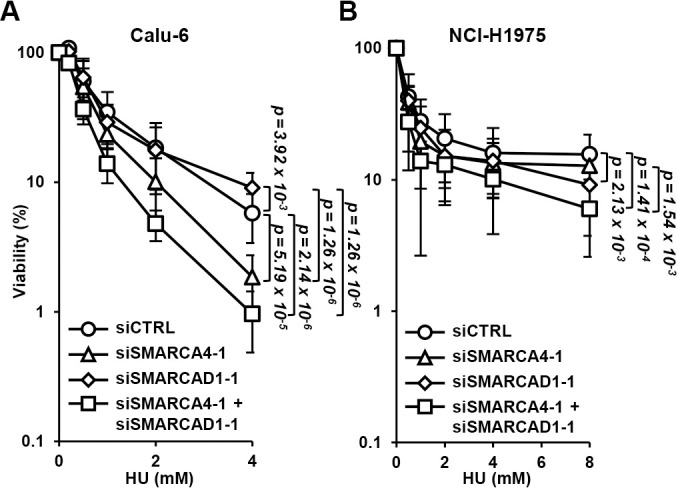
Redundant functions of SMARCA4 and SMARCAD1 to overcome replication stress Colony formation assay findings with (A) Calu-6 and (B) NCI-H1975 cells. Quantitative results relative to the untreated condition are shown. Colonies were counted and the results are shown as the mean±SD of three or more independent experiments.

Finally, we examined the contributions to HU sensitivity of SMARCA4 and SMARCAD1 together and separately. Clonogenic survival assay results showed that SMARCA4 depletion increased HU sensitivity in Calu-6 cells, whereas SMARCAD1 depletion did not; sensitivity was not increased by double knockdown compared with depletion of only SMARCA4 (Figure 5A). In NCI-H1975 cells, SMARCAD1 depletion increased HU sensitivity, while SMARCA4 depletion did not; double knockdown did not increase sensitivity compared with depletion of only SMARCAD1 (Figure 5B). This suggests that either SMARCA4 or SMARCAD1 is required in these cell lines for releasing replication stress and that SMARCAD1 may function as a back-up factor for SMARCA4.

## Discussion

**Figure 6:**
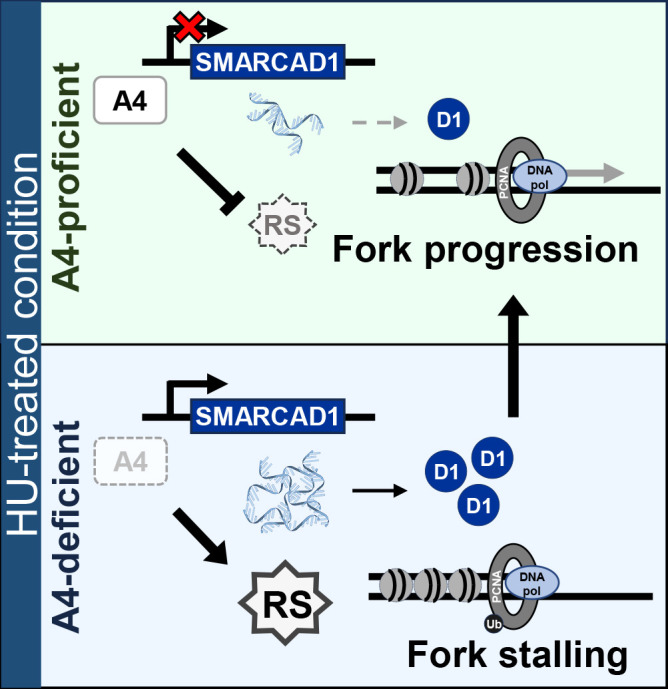
Proposed mechanism for the redundant functions of SMARCA4 and SMARCAD1 to maintain fork progression under replication stress in non-small cell lung cancer Replication stress is increased in the presence of HU. Under SMARCA4-proficient conditions, SMARCA4 reduces replication stress and allows fork progression, while SMARCAD1 transcription is suppressed by SMARCA4. Under SMARCA4-deficient conditions, replication forks are stalled and SMARCAD1 transcription is upregulated. Increased SMARCAD1 may be recruited at the stalled forks and rescue fork progression processes. RS: HU-induced replication stress, A4: SMARCA4, D1: SMARCAD1, Ub: ubiquitin, DNA pol: replicative DNA polymerase.

Our results indicate the SMARCA4-SMARCAD1 axis as a novel mechanism for the toleration of NSCLC cells to replication stress. Under SMARCA4-deficient conditions, SMARCAD1 may be recruited at stalled replication forks to support fork progression (Figure 6). Our findings also revealed the SMARCA4-SMARCAD1 axis in at least two (Calu-6 and NCI-H1975) of four NSCLC cell lines (Figure 3A; Figure S3B–E) and its functions in cancer cell survival. Cancer diversity in patients and single tumor heterogeneity often results in resistance to cancer treatments.^47,48^ Thus, elucidation and understanding of the underlying pathways may provide important information for future therapeutic strategies.

While previous studies have shown that SMARCAD1 expression is often upregulated in association with cancer,^13–16^ details regarding its functional interaction with other proteins to promote carcinogenesis remain largely unknown. Our results showed that *SMARCAD1* mRNA was increased in SMARCA4-depleted cells treated with HU (Figure 2B). We speculate that SMARCA4 binding of the transcriptional regulatory region of *SMARCAD1* is decreased by replication stress, suggesting that SMARCA4 is a transcriptional suppressor of SMARCAD1.

SMARCAD1 exhibits multiple roles in the progression of DNA replication, such as fine control of PCNA for replication tolerance, maintenance of heterochromatin inheritance, histone modifications, and R-loop suppression.^33–36^ SMARCAD1 is also required for histone eviction for post-replicative correction of replication errors.^12^ Notably, these functions largely overlap with those of SMARCA4. It is possible that in at least some NSCLC cell lines, SMARCAD1 expression regulated by SMARCA4 serves as a back-up system to release replication stress.

Regarding the observed upregulation of *SMARCAD1* mRNA in SMARCA4-depleted cells, we cannot exclude the possibility that the absence of SMARCA4 has an influence on *SMARCAD1* mRNA stability. We also found that SMARCAD1 protein levels were upregulated in SMARCA4-depleted cells in the absence of HU (Figure 3A; Figure S3B), whereas no upregulation of *SMARCAD1* mRNA was observed (Figure 2B; Figure S2), suggesting that a post-translational regulation mechanism may also be present. Further investigation will be required to elucidate the relationship of the SMARCA4-SMARCAD1 axis with replication stress.

SMARCA4 has recently been investigated as a promising therapeutic target for cancer. Mota et al. reported that the drug AU-15530, which degrades SMARCA4, effectively suppressed proliferation of SMARCA4-high diffuse intrinsic pontine gliomas.^49^ Other studies showed that SMARCA4-deficient lung adenocarcinoma cells were selectively sensitized to an ATR inhibitor because of the accumulation of replication stress induced by the loss of SMARCA4.^41,50^ Further research is required to explore the possibility of the SMARCA4-SMARCAD1 axis providing potential therapeutic targets for cancer.

## Figures and Tables

**Figure 4:**
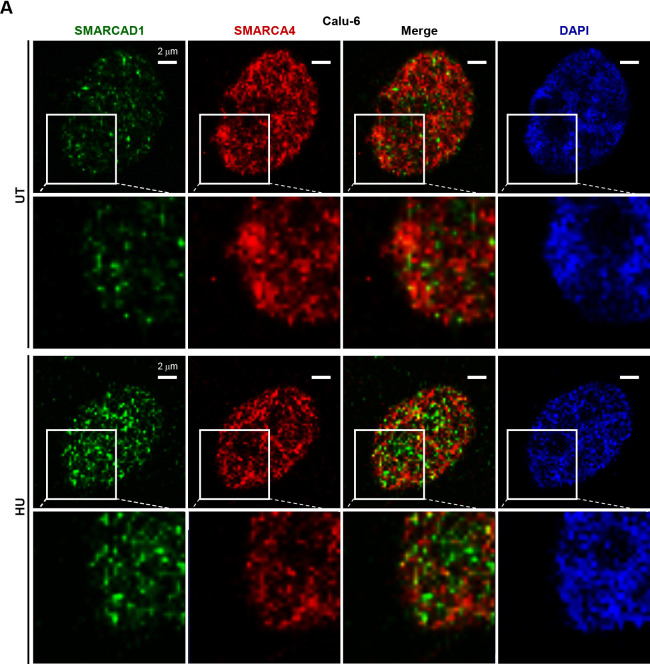
SMARCAD1 signaling in stalled forks increased by SMARCA4 depletion (A) Immunofluorescent staining of SMARCA4 (red) and SMARCAD1 (green) in Calu-6 cells. (B) Depletion of SMARCA4 enhanced co-localization of SMARCAD1 and RPA. Immunofluorescent staining of SMARCAD1 (green) and RPA (red) in Calu-6 cells was performed under SMARCA4 knockdown conditions. Cells were pre-treated with 1 mM HU (HU) or untreated (UT) for 24 hours. Experiments were performed twice, with similar results obtained. Scale bar, 2 μm.

**Figure 4:**
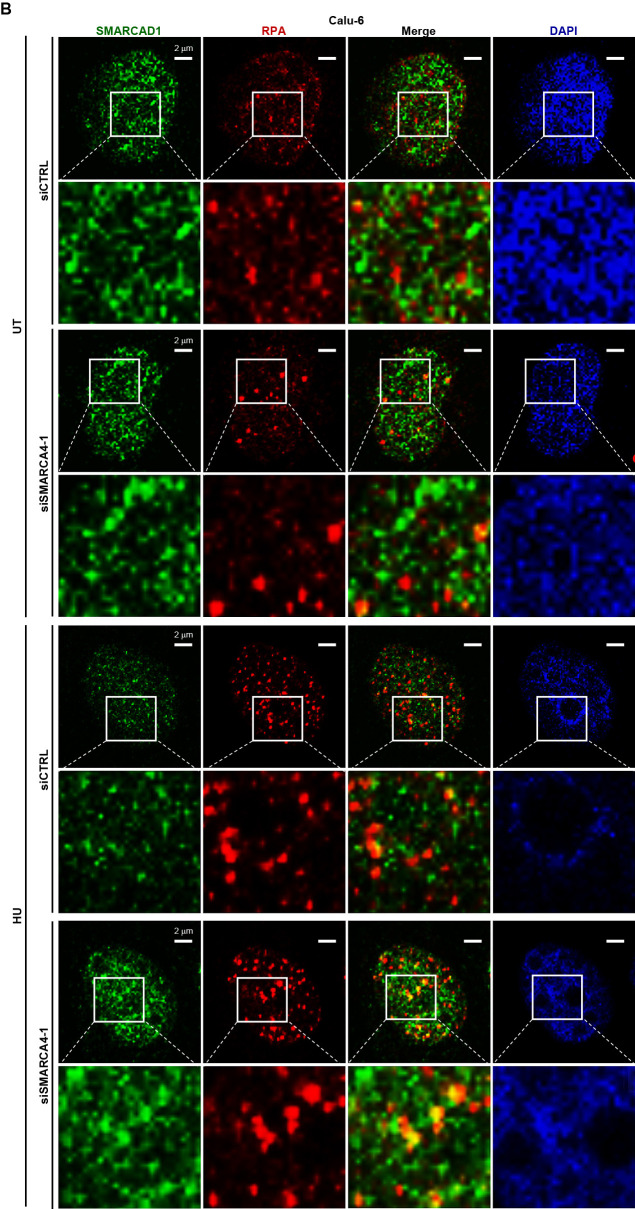
(cont.)
