# Supplementary material for: SMARCA4 regulates SMARCAD1 expression for toleration of replication stress in non-small cell lung cancer
Source: Fujita Med J. 2025 Nov 5;12(1):40–9. doi: 10.20407/fmj.2025-017 (PMC12865287; doi:10.20407/fmj.2025-017)
Supplement: Supplementary file 1 — Supplementary Materials [file fmj-12-040-s001.pdf]

Figure S1

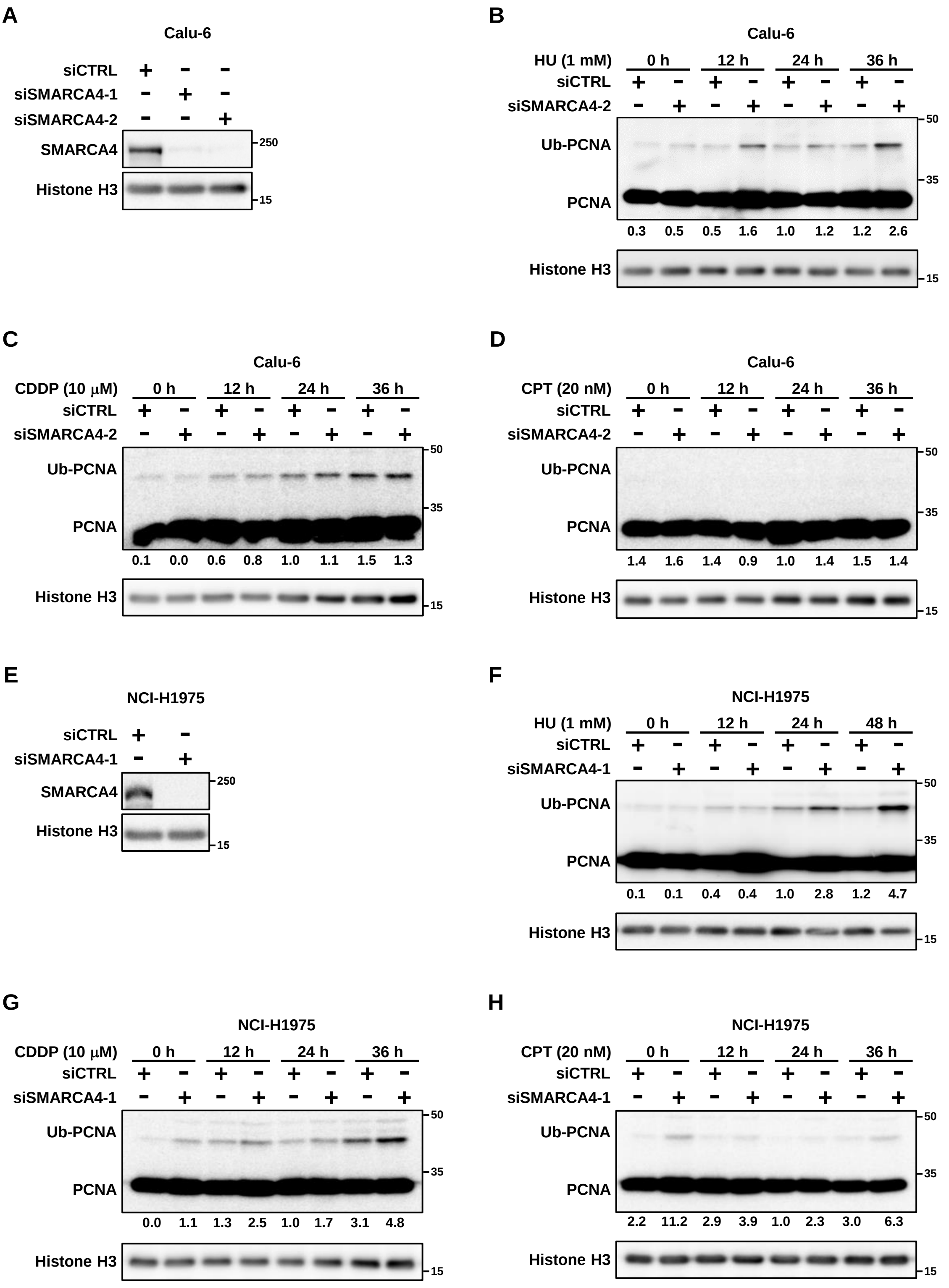

A
Calu-6
siCTRL + - -
siSMARCA4-1 - + -
siSMARCA4-2 - - +
SMARCA4
Histone H3
250
15
B
Calu-6
HU (1 mM) 0 h 12 h 24 h 36 h
siCTRL + - + - + - + -
siSMARCA4-2 - + - + - + - +
Ub-PCNA
PCNA
0.3 0.5 0.5 1.6 1.0 1.2 1.2 2.6
Histone H3
50
35
15
C
Calu-6
CDDP (10 μM) 0 h 12 h 24 h 36 h
siCTRL + - + - + - + -
siSMARCA4-2 - + - + - + - +
Ub-PCNA
PCNA
0.1 0.0 0.6 0.8 1.0 1.1 1.5 1.3
Histone H3
50
35
15
D
Calu-6
CPT (20 nM) 0 h 12 h 24 h 36 h
siCTRL + - + - + - + -
siSMARCA4-2 - + - + - + - +
Ub-PCNA
PCNA
1.4 1.6 1.4 0.9 1.0 1.4 1.5 1.4
Histone H3
50
35
15
E
NCI-H1975
siCTRL + -
siSMARCA4-1 - +
SMARCA4
Histone H3
250
15
F
NCI-H1975
HU (1 mM) 0 h 12 h 24 h 48 h
siCTRL + - + - + - + -
siSMARCA4-1 - + - + - + - +
Ub-PCNA
PCNA
0.1 0.1 0.4 0.4 1.0 2.8 1.2 4.7
Histone H3
50
35
15
G
NCI-H1975
CDDP (10 μM) 0 h 12 h 24 h 36 h
siCTRL + - + - + - + -
siSMARCA4-1 - + - + - + - +
Ub-PCNA
PCNA
0.0 1.1 1.3 2.5 1.0 1.7 3.1 4.8
Histone H3
50
35
15
H
NCI-H1975
CPT (20 nM) 0 h 12 h 24 h 36 h
siCTRL + - + - + - + -
siSMARCA4-1 - + - + - + - +
Ub-PCNA
PCNA
2.2 11.2 2.9 3.9 1.0 2.3 3.0 6.3
Histone H3
50
35
15

Figure S2

A

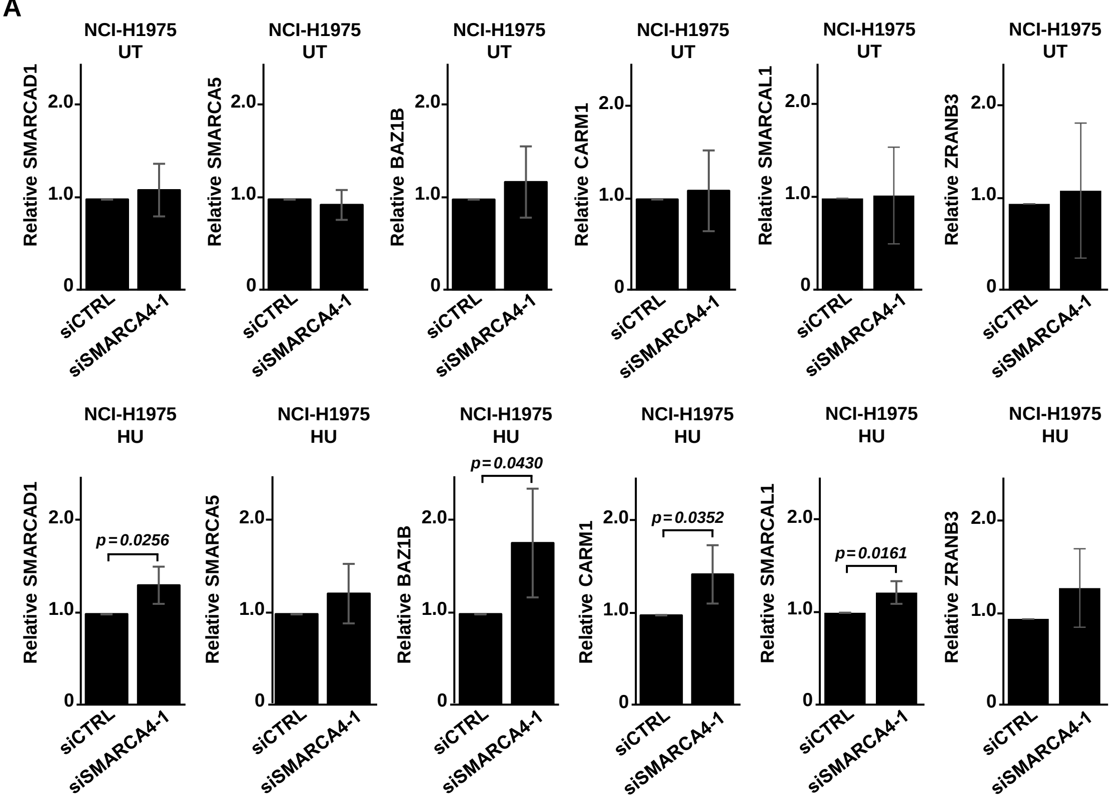
NCI-H1975
UT
Relative SMARCAD1
Relative SMARCA5
Relative BAZ1B
Relative CARM1
Relative SMARCAL1
Relative ZRANB3
2.0
1.0
0
siCTRL
siSMARCA4-1
NCI-H1975
HU
p=0.0256
p=0.0430
p=0.0352
p=0.0161

B

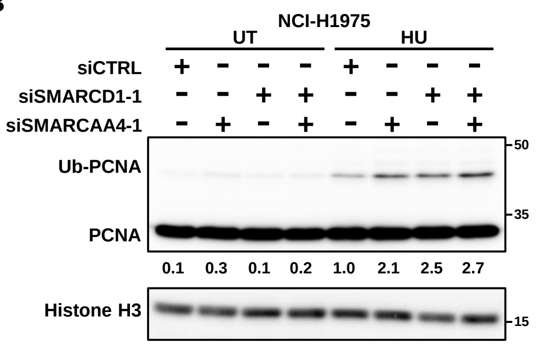
NCI-H1975
UT
HU
siCTRL + − − − + − − −
siSMARCD1-1 − − + + − − + +
siSMARCAA4-1 − + − + − + − +
Ub-PCNA
PCNA
50
35
0.1 0.3 0.1 0.2 1.0 2.1 2.5 2.7
Histone H3
15

Figure S3

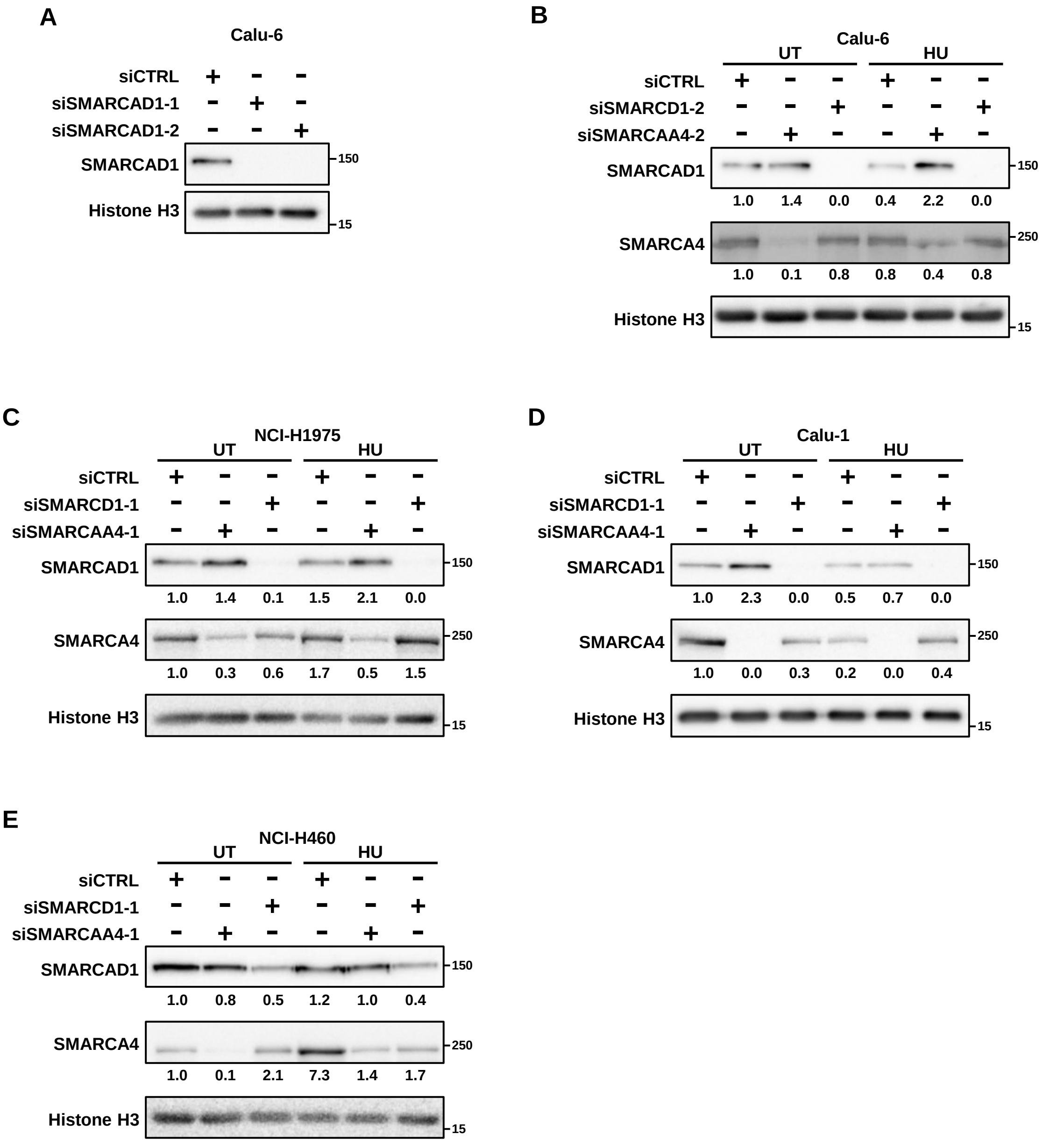
A
Calu-6
siCTRL + − −
siSMARCAD1-1 − + −
siSMARCAD1-2 − − +
SMARCAD1
Histone H3
150
15
B
Calu-6
UT
HU
siCTRL + − − + − −
siSMARCD1-2 − − + − − +
siSMARCAA4-2 − + − − + −
SMARCAD1
1.0 1.4 0.0 0.4 2.2 0.0
SMARCA4
1.0 0.1 0.8 0.8 0.4 0.8
Histone H3
150
250
15
C
NCI-H1975
UT
HU
siCTRL + − − + − −
siSMARCD1-1 − − + − − +
siSMARCAA4-1 − + − − + −
SMARCAD1
1.0 1.4 0.1 1.5 2.1 0.0
SMARCA4
1.0 0.3 0.6 1.7 0.5 1.5
Histone H3
150
250
15
D
Calu-1
UT
HU
siCTRL + − − + − −
siSMARCD1-1 − − + − − +
siSMARCAA4-1 − + − − + −
SMARCAD1
1.0 2.3 0.0 0.5 0.7 0.0
SMARCA4
1.0 0.0 0.3 0.2 0.0 0.4
Histone H3
150
250
15
E
NCI-H460
UT
HU
siCTRL + − − + − −
siSMARCD1-1 − − + − − +
siSMARCAA4-1 − + − − + −
SMARCAD1
1.0 0.8 0.5 1.2 1.0 0.4
SMARCA4
1.0 0.1 2.1 7.3 1.4 1.7
Histone H3
150
250
15

Figure S4

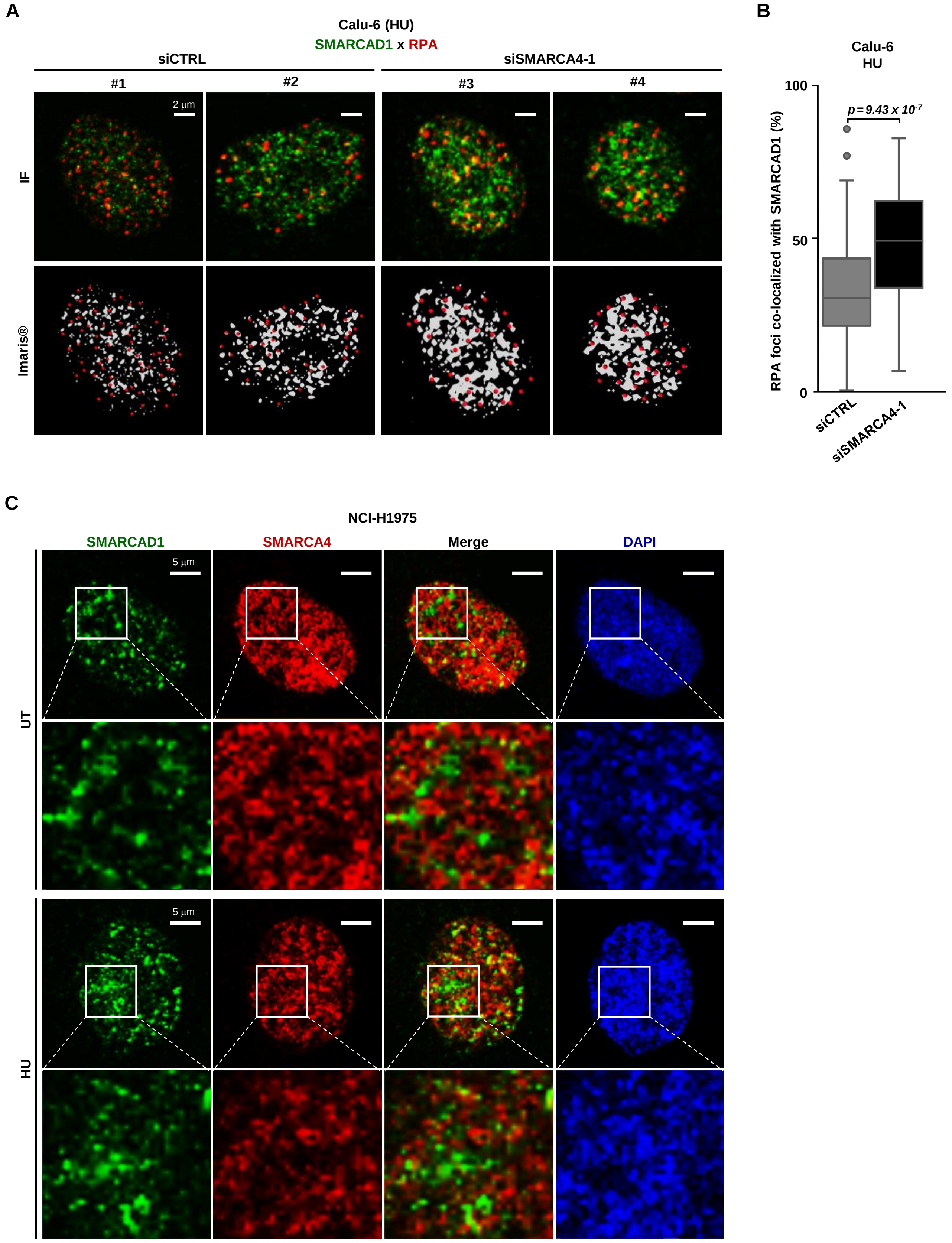
A
Calu-6 (HU)
SMARCAD1 x RPA
siCTRL
siSMARCA4-1
#1
#2
#3
#4
2 µm
IF
Imaris®
B
Calu-6
HU
100
50
0
p = 9.43 x 10-7
RPA foci co-localized with SMARCAD1 (%)
siCTRL
siSMARCA4-1
C
NCI-H1975
SMARCAD1
SMARCA4
Merge
DAPI
5 µm
UT
HU

Figure S4 (cont.)

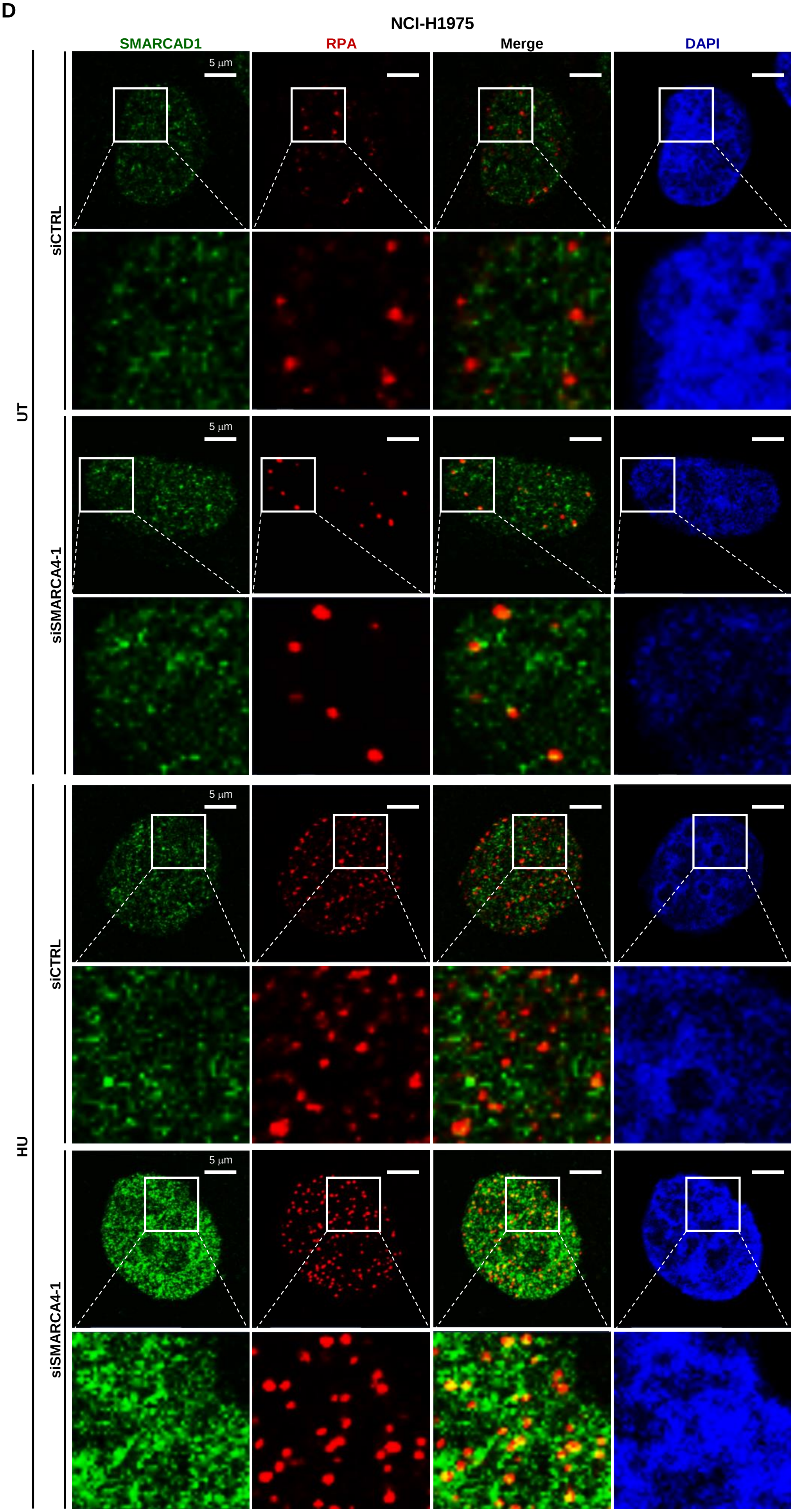

Supplementary figure legends

Figure S1. PCNA ubiquitination levels in SMARCA4 knockdown cells.

(**A**) Knockdown efficiency of siSMARCA4-1 and siSMARCA4-2 in Calu-6 cells shown by immunoblotting. (**B–D**) Calu-6 cells treated with siCTRL or siSMARCA4-2 were analyzed for PCNA ubiquitination (Ub-PCNA) with or without treatment with 1 mM hydroxyurea (HU) (**B**), 10 µM cisplatin (CDDP) (**C**), or 20 nM camptothecin (CPT) (**D**). (**E**) Knockdown efficiency of siSMARCA4-1 in NCI-H1975 cells. (**F–H**) NCI-H1975 cells treated with siCTRL or siSMARCA4-1 were analyzed for PCNA ubiquitination (Ub-PCNA) following treatment with 1 mM HU (**F**), 10 µM CDDP (**G**), or 20 nM CPT (**H**). Relative values to histone H3 are indicated.

Figure S2. mRNA levels of six candidate genes, *SMARCAD1*, *SMARCA5*, *BAZ1B*, *CARM1*, *SMARCAL1*, and *ZRANB3*, in NCI-H1975 cells.

(**A**) mRNA levels in cells treated with 1 mM HU (HU) or untreated (UT) cells were determined using qPCR. *P*-values were calculated from five independent experiments. (**B**) NCI-H1975 cells treated with siCTRL, siSMARCAD1-1, and/or siSMARCA4-1 with or without treatment with 1 mM HU were analyzed for PCNA ubiquitination (Ub-PCNA). Relative values to histone H3 are indicated.

Figure S3. SMARCA4 and SMARCAD1 protein levels in NSCLC cell lines.

(**A**) Knockdown efficiency of siSMARCAD1-1 and siSMARCAD1-2 in Calu-6 cells shown by immunoblotting. (**B**) SMARCA4 depletion in Calu-6 cells using the second set of siRNA resulted in upregulation of SMARCAD1 under treatment with 1 mM HU (HU) and untreated (UT) conditions. (**C–E**) Effect of SMARCA4 depletion in NCI-H1975

(**C**), Calu-1 (**D**), and NCI-H460 (**E**) cells. Relative values to histone H3 are indicated. All experiments were performed twice, with similar results obtained.

Figure S4. Immunofluorescent analysis of SMARCAD1 localization in stalled forks.

(**A**) Co-localization of SMARCAD1 and RPA. Representative images, generated by Imaris, version 9.8.2, show surface rendering of SMARCAD1 (white) and center of fluorescence intensity of RPA (red). Scale bar, 2 µm. (**B**) The percentage of RPA co-localized with SMARCAD1 was calculated using at least 60 cells. Similar results were obtained in two independent experiments. (**C**) Immunofluorescence of SMARCA4 (red) with SMARCAD1 (green) in NCI-H1975 cells. (**D**) Depletion of SMARCA4 enhanced co-localization of SMARCAD1 and RPA. immunofluorescent staining of SMARCAD1 (green) and RPA (red) was performed in NCI-H1975 cells with SMARCA4 knockdown. Cells were treated with 1 mM HU (HU) or untreated (UT) for 24 h. Experiments were performed twice, with similar results obtained. (**C, D**) Scale bar, 5 µm.

Table S1. List of antibodies and chemicals used in this study

Target	Mono/polyclonal	Clone/reference	Antibody raised in	Source	Dilution/ concentration for IB	Dilution/ concentration for IF	Amount of antibody for ChIP
SMARCA4	monoclonal	Ab110641	rabbit	Abcam	1:50 000	1:50 000	4 μg
SMARCA4	monoclonal	sc17796	mouse	Santa Cruz Cartel	-	1:500	-
SMARCAD1	polyclonal	12458	rabbit	Cell Signaling Technology	1:1 000	1:200	-
PCNA	monoclonal	PC10/sc2027	mouse	Santa Cruz Cartel	1:1 000	1:5 000	-
Histone H3	polyclonal	ab1791	rabbit	Abcam	1:50 000	-	-
RPA32/RPA2	monoclonal	ab2175	mouse	Abcam	-	1:400	-
Normal rabbit IgG	-	sc2027	rabbit	Santa Cruz Cartel	-	-	4 μg
Anti-mouse IgG conjugated with HRP		7076	horse	Cell Signaling Technology	1:1 000	-	-
Anti-rabbit IgG conjugated with HRP		7074s	goat	Cell Signaling Technology	1:1 000	-	-
mouse IgG conjugated with Alexa Fluor 488		A21202	donkey	Invitrogen	-	1:480	-
mouse IgG conjugated with Alexa Fluor 568		A10037	donkey	Invitrogen	-	1:480	-
rabbit IgG conjugated with Alexa Fluor 488		A21206	donkey	Invitrogen	-	1:480	-
rabbit IgG conjugated with Alexa Fluor 568		A10042	donkey	Invitrogen	-	1:480	-
Hydroxyurea (HU)	-	-	-	Tokyo Chemical Industry	1 mM	1 mM	-
Cisplatin (CDDP)	-	-	-	FUJIFILM Wako Pure Chemical Corporation	10 μM	-	-
Camptothecin (CPT)	-	-	-	Tokyo Chemical Industry	20 nM	-	-

Minus signs indicate that the antibody was not used for this application in this study. IB, immunoblotting; IF, immunofluorescent staining; ChIP, chromatin immunoprecipitation.

Table S2. List of siRNA and primers used in this study

Name	Supplier	Sequence (sense 5'-3') or siRNA ID from supplier
**siRNA**		
siCTRL	SIGMA	SIC002, MISSION® siRNA Universal Negative Control #2
siSMARCA4-1	SIGMA	SASI_Hs01_00019362
siSMARCA4-2	SIGMA	SASI_Hs02_00334991
siSMARCAD1-1	SIGMA	SASI_Hs02_00331176
siSMARCAD1-2	SIGMA	SASI_Hs02_00331177
**primer for qPCR**		
SMARCAD1F [1]	SIGMA (custom)	CTACCATGGCACGTAGAAATG
SMARCAD1R [1]	SIGMA (custom)	CACTTCTTTGTGGAAAAAGTTCC
SMARCA5F [2]	SIGMA (custom)	AACTTACTATCCGTTGGCGATT
SMARCA5R [2]	SIGMA (custom)	GGTTGCTTTGGAGCTTTCTG
BAZ1BF [3]	SIGMA (custom)	CCTCGCAGTAAGAAAGCAAAC
BAZ1BR [3]	SIGMA (custom)	ACTCATCCAGCTCCTTTTGAC
CARM1F2 [4]	SIGMA (custom)	GGCCACAACAACCTGATTCC
CARM1R [4]	SIGMA (custom)	TGGCTGTTGACTGCATAGTG
SMARCAL1F [5]	SIGMA (custom)	CAGAGGCAGACCTTTCTGAAG
SMARCAL1R [5]	SIGMA (custom)	CGGCCTCCTTTGGCTATG
ZRANB3F3 [6]	SIGMA (custom)	AGCCAGACGAGCCATTCTTC
ZRANB3R3 [6]	SIGMA (custom)	CTTTTTGCATAGTCGGTCCATCT
18SF	SIGMA (custom)	AATCAGGGTTCGATTCCGGA
18SR	SIGMA (custom)	CCAAGATCCAACTACGAGCT
**primer for chromatin immunoprecipitation (ChIP) assay**		
D1_set1F	SIGMA (custom)	CAGGTCCTTTCTCGAGGCAG
D1_set1R	SIGMA (custom)	GACCCTCATGCAACCTCCTC

References

[1] Zafari, V. *et al.* Regulatory Effect of let-7f Transfection in Non-Small Cell Lung Cancer on its Candidate Target Genes. *Iran Biomed J* **26**, 209-218, doi:10.52547/ibjhy.26.3.209 (2022).

[2] Zikmund, T. *et al.* Loss of ISWI ATPase SMARCA5 (SNF2H) in Acute Myeloid Leukemia Cells Inhibits Proliferation and Chromatid Cohesion. *Int J Mol Sci* **21**, doi:10.3390/ijms21062073 (2020).

[3] Zanella, M. *et al.* Dosage analysis of the 7q11.23 Williams region identifies BAZ1B as a major human gene patterning the modern human face and underlying self-domestication. *Sci Adv* **5**, eaaw7908, doi:10.1126/sciadv.aaw7908 (2019).

[4] Shlensky, D. *et al.* Differential CARM1 Isoform Expression in Subcellular Compartments and among Malignant and Benign Breast Tumors. *PLoS One* **10**, e0128143, doi:10.1371/journal.pone.0128143 (2015).

[5] Quinet, A. *et al.* PRIMPOL-Mediated Adaptive Response Suppresses Replication Fork Reversal in BRCA-Deficient Cells. *Mol Cell* **77**, 461-474.e469, doi:10.1016/j.molcel.2019.10.008 (2020).

[6] Rakesh, R. *et al.* Altering mammalian transcription networking with ADAADi: An inhibitor of ATP-dependent chromatin remodeling. *PLoS One* **16**, e0251354, doi:10.1371/journal.pone.0251354 (2021).

Table S3. List of “nuclear replication fork” genes in the cellular component shown in Gene Ontology resource (GOCC: nuclear replication fork, 41 genes)

List of genes associated with the term "nuclear replication fork" in the cellular component shown in Gene Ontology from AmiGO 2, version 2.5.17 (https://amigo.soybase.org/amigo/amigo/landing accessed on 2024-01-19).

BAZ1B	*BCAS2*	*CAMSAP3*	*CARM1*	*CDC5L*	*EME1*	*EME2*	*ERCC5*
ETAA1	*HELB*	*MCM10*	*MCM3*	*MMS22L*	*MUS81*	*PARP1*	*PCNA*
PLRG1	*POLA1*	*POLA2*	*POLD1*	*POLD2*	*POLD3*	*POLD4*	*PRIM1*
PRIM2	*PRPF19*	*RPA1*	*RPA2*	*RPA3*	*RPA4*	*SMARCA5*	*SMARCAD1*
SMARCAL1	*TIMELESS*	*TIPIN*	*TONSL*	*TREX1*	*WDHD1*	*XPA*	*ZMIZ2*
ZRANB3							

Table S4. List of "chromatin organization and chromatin remodeling" genes in the biological process shown in Gene Ontology resource (GOBP: chromatin organization and chromatin remodeling, 650 genes)

List of genes associated with the term "chromatin organization and chromatin remodeling" in the biological process shown in Gene Ontology from AmiGO 2, version 2.5.17 (https://amigo.soybase.org/amigo/amigo/landing accessed on 2024-01-19).

ACTB	*ACTL6A*	*ACTL6B*	*ACTR5*	*ACTR6*	*ACTR8*
AICDA	*ALKBH4*	*ANP32B*	*ANTKMT*	*APLF*	*APOBEC1*
ARB2A	*ARB2BP*	*ARID1A*	*ARID1B*	*ARID2*	*ARID4A*
ARID4B	*ARRB1*	*ASF1A*	*ASF1B*	*ASH1L*	*ASH2L*
ASIP	*ATF2*	*ATF7IP*	*ATF7IP2*	*ATM*	*ATPSCKMT*
ATR	*ATRX*	*AURKA*	*AXIN1*	*BABAM1*	*BAHD1*
BAP1	*BAZ1A*	*BAZ1B*	*BAZ2A*	*BAZ2B*	*BCL6*
BCL7A	*BCL7B*	*BCL7C*	*BCOR*	*BEND3*	*BICRA*
BICRAL	*BMI1*	*BPTF*	*BRCA1*	*BRCA2*	*BRCC3*
BRD1	*BRD2*	*BRD7*	*BRD9*	*BRDT*	*BRPF1*
BRPF3	*BTAF1*	*BUB1*	*C6orf89*	*CABIN1*	*CARM1*
CBX3	*CDK1*	*CDK2*	*CDK9*	*CDKN1C*	*CDY1*
CDY2A	*CDYL*	*CECR2*	*cecr2-smarca1_human*	*CENPA*	*CENPI*
CENPN	*CENPP*	*CENPV*	*CENPW*	*CFDP1*	*CGGBP1*
CHAF1A	*CHAF1B*	*CHD1*	*CHD1L*	*CHD2*	*CHD3*
CHD4	*CHD5*	*CHD6*	*CHD7*	*CHD8*	*CHD9*
CHEK1	*chrac_human*	*CHRAC1*	*CHTOP*	*CLOCK*	*COPRS*
CREBBP	*CREBZF*	*CTCF*	*CTCFL*	*CTR9*	*cyclina2_cdk2_human*
DAXX	*DCAF1*	*DCAF13*	*DDB1*	*DDX21*	*DDX23*
DDX4	*DEK*	*DIRAS3*	*DMAP1*	*DMRTC2*	*DNAJC9*
DNMT1	*DNMT3A*	*DNMT3L*	*DOT1L*	*DPF1*	*DPF2*
DPF3	*DPPA3*	*DPY30*	*DTX3L*	*DYRK1A*	*EED*
EEF1AKMT1	*EEF1AKMT2*	*EEF1AKMT3*	*EEF2KMT*	*EHMT1*	*EHMT2*
EOMES	*EP300*	*EP400*	*EPC1*	*EPOP*	*ERCC6*
ERCC6L	*ERCC6L2*	*ESR1*	*EYA1*	*EYA2*	*EYA3*
EZH1	*EZH2*	*EZHIP*	*fact_human*	*FAM47E*	*FBL*
FBLL1	*FBXL19*	*FOXA1*	*FOXP3*	*GATA3*	*g-atac_human*
GATAD1	*GATAD2A*	*GATAD2B*	*GLMN*	*GLYR1*	*GPX1*
GRWD1	*GSK3A*	*GTF2B*	*GTF3C4*	*H1-0*	*H1-1*
H1-10	*H1-2*	*H1-3*	*H1-4*	*H1-5*	*H1-6*
H1-8	*H1-9P*	*H2AB1*	*H2AB2*	*H2AC1*	*H2AC11*

Table S4. (cont.)

H2AC12	*H2AC14*	*H2AC18*	*H2AC20*	*H2AC21*	*H2AC25*
H2AC4	*H2AC6*	*H2AC7*	*H2AJ*	*H2AL3*	*H2AP*
H2AX	*H2AZ1*	*H2AZ2*	*H2BC1*	*H2BC10*	*H2BC11*
H2BC13	*H2BC14*	*H2BC15*	*H2BC17*	*H2BC21*	*H2BC3*
H2BC9	*H3-3A*	*H3-4*	*H3C1*	*H3C13*	*H4C1*
H4C7	*HASPIN*	*HAT1*	*hbo1-4.1 human*	*hbo1-4.2_ human*	*hbo1-4.3_ human*
hbo1-5.1_ human	*hbo1-5.2_ human*	*hbo1-5.3_ human*	*HCFC1*	*HCFC2*	*HDAC1*
HDAC10	*HDAC11*	*HDAC2*	*HDAC3*	*HDAC4*	*HDAC5*
HDAC6	*HDAC7*	*HDAC8*	*HDAC9*	*HDGF*	*HDGFL1*
HDGFL2	*HDGFL3*	*HELLS*	*HIPK2*	*HIPK4*	*HIRA*
HJURP	*HLTF*	*HMGA1*	*HMGA2*	*HMGB1*	*HMGB2*
HNRNPC	*HNRNPU*	*HP1BP3*	*HPF1*	*HR*	*HUWE1*
IFI16	*IGF2*	*ING3*	*ING4*	*INO80*	*ino80_human-1*
INO80B	*INO80C*	*INO80D*	*INO80E*	*IRF4*	*ITGB3BP*
IWS1	*JADE1*	*JADE2*	*JAK2*	*JARID2*	*JDP2*
JMJD1C	*JMJD6*	*KAT14*	*KAT2A*	*KAT2B*	*KAT5*
KAT6A	*KAT6B*	*KAT7*	*KAT8*	*KDM1A*	*KDM1B*
KDM2A	*KDM2B*	*KDM3A*	*KDM3B*	*KDM4A*	*KDM4B*
KDM4C	*KDM4D*	*KDM4E*	*KDM4F*	*KDM5A*	*KDM5B*
KDM5C	*KDM5D*	*KDM6A*	*KDM6B*	*KDM7A*	*KDM8*
kiaa0409-suv39h1_human	*KLF2*	*KMT2A*	*KMT2B*	*KMT2C*	*KMT2D*
KMT2E	*KMT5A*	*KMT5B*	*KMT5C*	*KPNA7*	*L3MBTL1*
L3MBTL3	*l3mbtl-cbx3_human*	*LHX2*	*LIN54*	*LMNA*	*LMNB1*
LMNB2	*LOXL2*	*LRIF1*	*MACROH2A1*	*MACROH2A2*	*MAEL*
MAP3K7	*MBD2*	*mbd2/nurd_human*	*MBD3*	*mbd3/nurd_human*	*MBD3L1*
MBD3L2	*MBD3L2B*	*MBD3L3*	*MBD3L4*	*MBD3L5*	*MCM2*
MCM3AP	*MCRS1*	*MEAF6*	*MECOM*	*MECP2*	*MEN1*
METTL21A	*METTL22*	*METTL23*	*METTL3*	*METTL4*	*MIER1*
MIER2	*MIER3*	*MIS18A*	*MORC1*	*MORC2*	*morf1_human*
moz1_human	*MPHOSPH8*	*msl_human*	*MSL1*	*MSL3*	*MTA1*
MTA2	*MTA3*	*MTF2*	*MTHFR*	*MYBBP1A*	*MYC*
MYD88	*MYOCD*	*MYSM1*	*N6AMT1*	*NAA40*	*NAA50*
NAA60	*NAP1L1*	*NAP1L2*	*NAP1L3*	*NAP1L4*	*NAP1L5*
NAP1L6P	*NASP*	*NCOA1*	*NCOA3*	*NDN*	*NFAT5*
NFE2	*NFKBIZ*	*NFRKB*	*NIBAN2*	*NIPBL*	*NOC2L*
NPM1	*NPM2*	*NPM3*	*NRDE2*	*NSD1*	*NSD2*
NSD3	*nsl_human*	*NTMT1*	*nua4_human*	*NUDT5*	*nurf_human*
OIP5	*PADI2*	*PADI4*	*PAK1*	*PARP2*	*PAX6*
PAXIP1	*PBRM1*	*PCGF1*	*PCGF2*	*PCGF3*	*PCGF5*

Table S4. (cont.)

PCGF6	*PCID2*	*PER1*	*PER2*	*PHB1*	*PHC1*
PHF1	*PHF10*	*PHF19*	*PHF2*	*PHF8*	*piccolo_nua4_human*
PICK1	*PIH1D1*	*PIK3CA*	*PIWIL1*	*PIWIL2*	*PKN1*
POLE3	*PPHLN1*	*PPM1D*	*PRDM13*	*PRDM14*	*PRDM16*
PRDM2	*PRDM6*	*PRDM7*	*PRDM8*	*PRDM9*	*PRIMPOL*
PRKAA1	*PRKAA2*	*PRKCA*	*PRKCB*	*PRKDC*	*PRMT1*
PRMT2	*PRMT5*	*PRMT6*	*PRMT7*	*PRMT8*	*PSIP1*
PWWP2A	*PWWP2B*	*RAD54B*	*RAD54L*	*RAD54L2*	*RB1*
RBBP4	*RBBP5*	*RBBP7*	*RBM14*	*RBM15*	*RBM15B*
RERE	*RESF1*	*REST*	*RIF1*	*RING1*	*RIOX1*
RIOX2	*RLIM*	*RNF168*	*RNF2*	*RNF20*	*RNF8*
RPS6KA4	*RPS6KA5*	*RRP8*	*RSBN1*	*RSF1*	*rsf1-smarca5_human*
RUVBL1	*RUVBL2*	*RYBP*	*saga-kat2a_human*	*SAMD1*	*SAMD7*
SART3	*SATB1*	*SATB2*	*SCMH1*	*SDR16C5*	*SET*
SETBP1	*SETD1A*	*SETD1B*	*SETD2*	*SETD3*	*SETD4*
SETD5	*SETD7*	*SETDB1*	*SETDB2*	*SETMAR*	*SETSIP*
SF3B1	*SFPQ*	*SGF29*	*SHPRH*	*SIN3A*	*SIRT1*
SIRT2	*SIRT3*	*SIRT4*	*SIRT6*	*SIRT7*	*SKP1*
SLFN11	*SMARCA1*	*SMARCA2*	*SMARCA4*	*SMARCA5*	*SMARCAD1*
SMARCAL1	*SMARCB1*	*SMARCC1*	*SMARCC2*	*SMARCD1*	*SMARCD2*
SMARCD3	*SMARCE1*	*SMCHD1*	*SMYD1*	*SMYD2*	*SMYD3*
SMYD5	*SNAI1*	*SOX9*	*SPHK2*	*SPI1*	*SPTY2D1*
SRCAP	*SRPK2*	*SS18*	*SSRP1*	*STPG4*	*SUDS3*
SUPT16H	*SUPT4H1*	*SUPT5H*	*SUPT6H*	*SUV39H1*	*SUV39H2*
SUZ12	*TADA2A*	*TADA2B*	*TAF1*	*TAF10*	*TAF1L*
TAF6L	*TAF9*	*TASOR*	*TASOR2*	*TASP1*	*TBR1*
TDG	*TDRD9*	*TET1*	*TET3*	*TEX15*	*TFAP2C*
tfiid_human	*tfiid-taf4bvariant_human*	*TFPT*	*TGM2*	*TNP1*	*TP53*
TP63	*TPR*	*TRIM27*	*TRIM28*	*TRIM37*	*TRIP12*
TRMT112	*TSPY1*	*TSPY10*	*TSPY2*	*TSPY26P*	*TSPY3*
TSPY4	*TSPY8*	*TSPY9*	*TSPYL1*	*TSPYL2*	*TSPYL4*
TSPYL5	*TSPYL6*	*TTF2*	*TTLL12*	*UBE2A*	*UBN1*
UBR2	*UBR5*	*UCHL5*	*UHRF1*	*UIMC1*	*USP15*
USP16	*USP21*	*USP22*	*USP3*	*USP36*	*USP49*
USP51	*USP7*	*UTY*	*VCPKMT*	*VPS72*	*VRK1*
WAC	*WBP2*	*WDR5*	*YEATS2*	*YEATS4*	*YTHDC1*
YY1	*ZBTB1*	*ZBTB7A*	*ZDBF2*	*ZFP57*	*ZMPSTE24*
ZNF274	*ZNF304*	*ZNF335*	*ZNF445*	*ZNF827*	*ZNFX1*
ZNHIT1	*ZRANB3*				
